# Evaluating the Effects of Non-Nutritive Sweeteners on Pigs: A Systematic Review

**DOI:** 10.3390/ani14203032

**Published:** 2024-10-19

**Authors:** Mariah R. Jansen, Kwangwook Kim

**Affiliations:** Department of Animal Science, Michigan State University, East Lansing, MI 48824, USA; jansenm2@msu.edu

**Keywords:** growth performance, gut health, non-nutritive sweetener, nutrition, pig, sugar substitutes, swine nutrition

## Abstract

Non-nutritive sweeteners (NNS) have been investigated for their potential to improve feed palatability and growth performance in pigs, although their use in swine production remains limited. This systematic review evaluates the effects of NNS on pigs, drawing from 18 studies published between 1990 and 2024. Following the PRISMA guidelines and using the PICOS framework, a total of 448 papers were initially identified, of which 18 met the inclusion criteria for review. The results are mixed: some studies suggest that NNS like stevioside, sucralose, and neotame may improve performance and reduce diarrhea, while others show limited or no effects. The impact of NNS on gut microbiota is similarly inconsistent, with some sweeteners promoting beneficial bacterial growth, while others show minimal changes in microbial diversity. This review emphasizes the need for more research to clarify the effects of NNS in pigs, particularly the mechanisms behind their influence on growth and gut health. Additionally, further studies are needed to determine optimal dosages and assess the long-term impacts of NNS on pig immune function and overall health. The findings highlight the current gaps in knowledge and suggest that more evidence is needed to understand the role of NNS in swine nutrition.

## Simple Summary

This systematic review examines the effects of non-nutritive sweeteners (NNS) on pigs, focusing on growth performance, feed preference, gut health, and other clinical indicators. Sweeteners such as stevia, sucralose, and neotame have been tested in various studies to evaluate their influence on swine production. Results show that NNS supplementation generally improves growth performance and feed intake in pigs, with some studies reporting reduced diarrhea rates and improved gut health. However, the effects of NNS on gut microbiota are inconsistent, with some sweeteners promoting beneficial bacteria growth while others show minimal changes in microbial diversity. Despite these outcomes, research on the long-term effects of NNS on gut health and the immune system remains limited. This review highlights the need for further studies to explore the mechanisms behind NNS effects, especially in diverse dietary and environmental conditions. Identifying optimal types and dosages of NNS, along with understanding their interactions with the gut microbiome, will be crucial in determining their role as a dietary supplement in swine production.

## 1. Introduction

Non-nutritive sweeteners (NNS), also known as high-intensity sweeteners or sugar substitutes, have minimal caloric value and are 10 to 1000 times sweeter than sucrose [1,2,3], which is the sweetener commonly found in foods like candy and soda [4]. The discovery of NNS began with saccharin in 1879, followed by significant advancements from the 1960s to the 1980s with the development of acesulfame-K, aspartame, sucralose, neotame, advantame, and steviol glycosides [5,6]. Their use surged in the 2000s, particularly in low-calorie foods and medications, with several sweeteners gaining approval from the FDA and the EU [7,8].

NNS have become increasingly popular in both human nutrition and livestock feeds. As their use expanded in livestock, it became essential to determine the preference and palatability of these sweeteners among different species. Various preference tests conducted on livestock such as cattle, sheep, and goats showed positive results [9,10,11]. In ruminants, particularly dairy cattle, NNS have been used to activate sweet taste receptors in the small intestine, potentially increasing glucose uptake and influencing rumen microbiota [12,13,14]. Studies on calves have also assessed the impact of these sweeteners on growth performance and feed preference during stress and production [15,16]. Similarly, in poultry, NNS have been tested for their effects on growth performance and feed preference, with research investigating their impact on intestinal morphology and immune responses to understand the mechanisms behind these improvements [17,18]. The intense sweetness of these sweeteners offered benefits such as increased feed palatability and intake, reduced inflammation, and positive changes to the gut microbiota, which positively impacted livestock health at a lower cost than sucrose [19,20,21,22]. Consequently, NNS became valuable feed additives to reduce calorie depletion and promote health during critical periods such as weaning [2].

Despite these positive results, the mechanisms underlying the benefits of NNS are not yet thoroughly understood. One potential mechanism is the activation of specific taste receptors by NNS, leading to the secretion of beneficial hormones that regulate appetite, glucose metabolism, and digestive processes [12,23,24]. Another mechanism involves the modification of gut microbiota, which can improve overall gut health and function [13,14,25]. Alterations in the gut microbiome can enhance nutrient absorption, boost immune responses, and reduce inflammation by promoting the growth of beneficial bacteria and inhibiting harmful pathogens. Additionally, NNS may influence the expression of genes related to metabolic pathways and immune function, further contributing to their positive effects on health [18].

However, minimal research has been conducted on the supplementation of NNS, particularly in pigs. While there is growing evidence of the benefits of NNS in other animals, specific studies on pigs are limited, leaving a gap in our understanding of how these sweeteners affect pig physiology, growth, and health. Investigating these aspects in pigs is essential to optimize their use and harness their full potential in improving livestock production.

Therefore, this systematic review aims to provide a comprehensive overview of data concerning feed preference, growth performance, health promotion, and the gut microbiome in pigs. It also seeks to understand the optimal use and underlying mechanisms of NNS supplementation, highlighting areas that require further investigation. By synthesizing existing research, this review aims to identify gaps in the current knowledge and propose directions for future studies to optimize the use of NNS in pig nutrition.

## 2. Materials and Methods

### 2.1. Study Protocol

The protocol of this systematic review was registered at the International Prospective Register of Systematic Reviews (PROSPERO) (ID: CRD42024518080 [26]). The systematic review was conducted in accordance with the Preferred Reporting Items for Systematic Reviews and Meta-analysis (PRISMA) guidelines.

### 2.2. Eligibility Criteria

The summary of the inclusion and exclusion criteria of the study characteristics, based on the PICOS framework (i.e., populations, interventions, comparators, outcomes of interest, and study designs) [27], is presented in Table 1. Peer-reviewed papers that were available in full-text and written in English were included. Review papers, abstracts, protocols, editorials, opinion pieces, and dissertations were excluded.

### 2.3. Information Sources and Search Strategy

The published research studies included in this review were found through searches in scientific databases, performed using the PubMed Advanced Search Builder, Scopus, Web of Science Core Collection, and AGRICOLA-USDA. A series of developed keywords utilizing Boolean search terms were used. A combination of keyword searches was employed to identify studies on non-nutritive sweeteners in pigs, and comprehensive search strategies for each database are provided in Table 2. The searches were completed in March 2024, with date limits applied to studies published between 1990 and 2024.

### 2.4. Study Selection

All retrieved references were imported into Zotero reference management software (version 6.0.37), and duplicates were initially removed. The remaining references were then imported into Covidence systematic review software [28], where additional duplicates were removed. Prior to article screening, two researchers (M.R.J. and K.K.) developed a procedure for title and abstract screening using 20 randomly selected papers. In the first phase of screening, M.R.J. and K.K. independently assessed all study titles and abstracts against the eligibility criteria in Table 1. The agreement in the abstract and title screening between the two reviewers was 84.8% (Cohen’s Kappa = 0.615). Then, a full-text review was performed. The agreement for full-text screening was 80.0% (Cohen’s Kappa = 0.636). Discrepancies at each stage were resolved through discussion with two reviewers.

### 2.5. Data Collection Process and Data Items

Based on the selected study criteria “Effects of Non-Nutritive Sweeteners in Pigs”, data extraction was performed. The data extraction forms were initially drafted by M.R.J. and discussed with K.K. Data extracted from each study included the following items: the names of the authors, the year of publication, and the country where the study was conducted; the number and species of animals used in the study; the mean age of the animals at the start of the experiment; the type and dosage of sweetener administered, along with the number of animals per treatment group; the total duration of the experiment; specific outcomes and parameters measured during the study, such as growth performance, feed intake, feed preference, gut health, and biochemical markers; and key findings from the study, including the effects of different sweeteners on the evaluated characteristics. These data items were systematically extracted to ensure consistency and comprehensiveness in capturing the relevant details of each study, facilitating a thorough comparison and synthesis of the results.

### 2.6. Quality Assessment and Risk of Bias

Two reviewers (M.R.J. and K.K.) independently evaluated the risk of bias of included studies using the SYRCLE’s risk of bias tool for animal experimental studies. The checklist comprises ten domains, categorized into six types of biases: sequence generation, baseline characteristics, and allocation concealment (selection bias); random housing and blinding of caregivers and/or investigators (performance bias); random outcome assessment and blinding of outcome assessors (detection bias); incomplete outcome data (attrition bias); selective outcome reporting (reporting bias); and other sources of bias (other). Each item in the tool was assessed as “low risk of bias”, “high risk of bias”, or “unclear risk of bias”. Disagreements between the reviewers were resolved through discussion.

### 2.7. Synthesis of Results

Given the heterogeneity in study outcomes, outcome measures, and trial designs, a qualitative evaluation and synthesis of the study results were performed. Consequently, a meta-analysis was not conducted, and publication bias was not assessed.

## 3. Results

### 3.1. Study Selection Process

**Figure 1:**
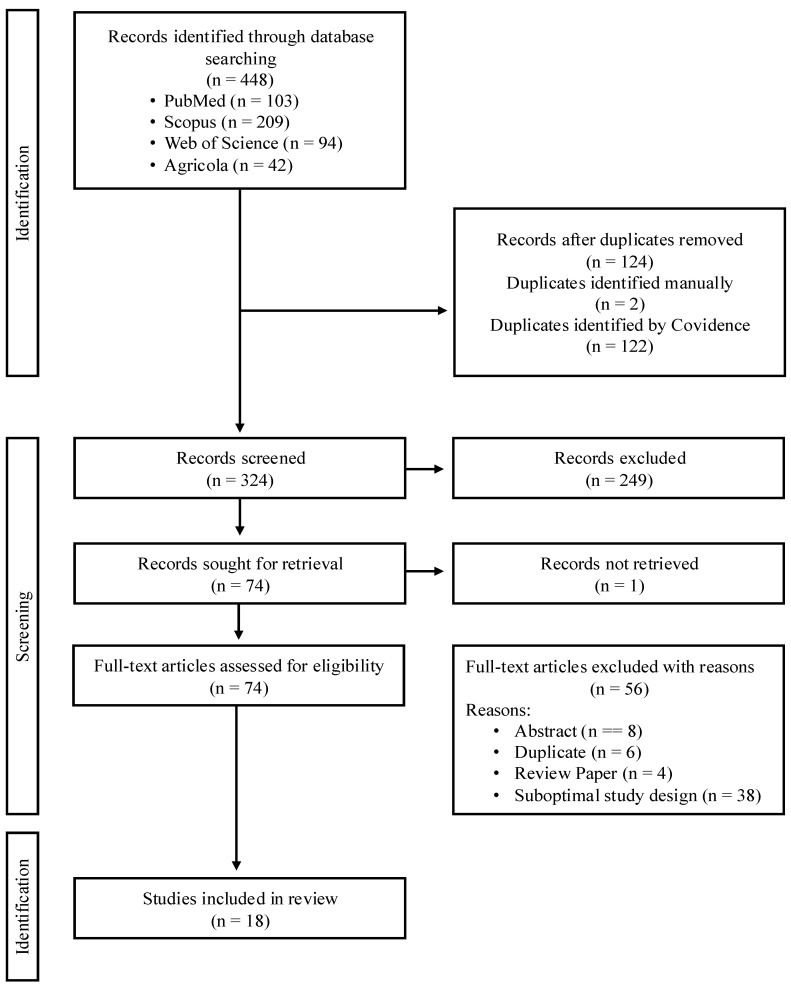
PRISMA flow diagram for the Covidence literature screening on non-nutritive sweetener supplementation in pigs.

Figure 1 shows the study selection process. The search yielded 448 references in total, from which 124 duplicates were removed. A total of 324 abstracts were then screened, among which 249 were judged ineligible, leaving 74 papers to be read in full text. In total, 18 papers met the eligibility criteria and were included.

### 3.2. Summary of Study Designs and Sample Characteristics

Table 3 provides details of the characteristics of each study. Four papers were published between 2020 and 2024, nine papers were published between 2010 and 2019, four papers were published between 2000 and 2009, and one paper was published before 2000. The total sample size ranged from 12 pigs [29] to 216 pigs [30,31].

Most of the studies used crossbred pigs as the sample population. These crossbreds included Large White/Landrace × Pietrain pigs [32], Great Yorkshire × Dutch Landrace × D-line [33], Landrace × Large White [34,35,36,37], Duroc × Landrace × Large White [30,31], Duroc × Landrace × Yorkshire [38,39,40], and Large White/Landrace × Large White [41]. Two studies experimented with purebred pigs, specifically Yorkshire [42] and Gloucestershire Old Spot pigs [22]. Four studies did not report the breed of pigs used [29,43,44,45].

Most studies used young pigs ranging from 21 to 28 days of age or weighing between 7.01 ± 0.3 to 9.05 ± 0.04 kg [22,30,31,32,33,35,36,37,38,39,42,43,45]. Other studies used pigs of different weights and ages, including those weighing approximately 23 kg [29], 68.08 ± 0.74 kg [40], and 34.1 ± 2.5 kg [41], as well as pigs aged between 2 to 4 months [44].

### 3.3. Summary of Non-Nutritive Sweetener Intervention and Evaluated Characteristics

Table 3 provides details of the non-nutritive sweetener interventions of each study. The studies used a variety of non-nutritive sweeteners across different experimental setups, including the variety of parameters to measure in each intervention. Aspartame, acesulfame-K, and cyclamate were added to the drinking water in two studies by Daly [36] and Glaser [43]. These studies measured the activation of the pig sweet taste receptor and conducted preference tests using the Richter-type drinking test. Alitame, Dulcin, Monellin, 5-Nitro-2-propoxy aniline (P-4000), Perillartine, and Thaumatin were added to the drinking water, and gustatory responses, preference, and Richter-type drinking tests were conducted [44]. Maltodextrin was added to both feed and water to measure feed and water consumption and was tested for two-choice drinking and feeding [41]. Neotame was included in the diet in different concentrations to assess the effects on feed intake, diet preference, growth performance, hematological and serum biochemical parameters, and histopathological parameters [31], as well as growth performance, nutrient digestibility, blood biochemical analysis, and fecal bacterial counts [38]. Various studies have tested saccharin in either feed or drinking water to evaluate different parameters: activation of the pig sweet taste receptor [37]; feed and water consumption, and two-choice drinking and feeding [41]; growth performance, feed preferences, nutrient digestibility, blood biochemicals, and fecal bacterial counts [38]; gustatory responses, preference test, Richter-type drinking test [44]; and expression of Na^+^/glucose co-transporter (SGLT1), and detection of gut hormones and sweet taste receptors [34]. A combination of saccharin and neotame in different doses was also added to the diet to measure growth performance, feed preferences, nutrient digestibility, blood biochemicals, and fecal bacterial counts [38]. Stevia or stevia residue was added to the diet in various concentrations to measure growth performance [42]; growth performance and feed preferences [32]; growth performance, carcass traits, meat quality, antioxidant capacity, and gut microbiota [40]; and growth performance, diarrhea rate, antioxidant capacity, intestinal health, and gut microbiota [39]. Stevioside was supplemented in the diet in different doses to assess growth performance and diarrhea incidence rate [30], as well as metabolism and absorption mechanisms in feces and blood [29]. Sucralose in various doses was added to the diet and drinking water to test the activation of the pig sweet taste receptor [37]; growth performance, feed intake, diet preference, hematological and serum parameters, organ index, and histopathological analysis [45]; and gustatory responses, preference, and Richter-type drinking test [44]. NHDC + saccharin (a combination of neohesperidin dihydrochalcone (NHDC) and saccharin) was added in various concentrations to the feed or drinking water to measure growth performance, feed intake per visit, latency time and total visits per day, number of visits with feed consumed, and fecal consistency [33]; mucosa-associated microbiota [22]; gut microbiota and cecal lactic acid concentration [35]; gut microbiota and cecal short-chain fatty acid concentration analysis [36]; and the expression of SGLT1, detection of gut hormones, and sweet taste receptors [34]. Sucrose or Rebaudioside A was added to the diet to measure growth performance [42], and growth performance and diarrhea incidence rate [30], respectively.

### 3.4. Summary of Quality Assessment and Risk of Bias

**Figure 2:**
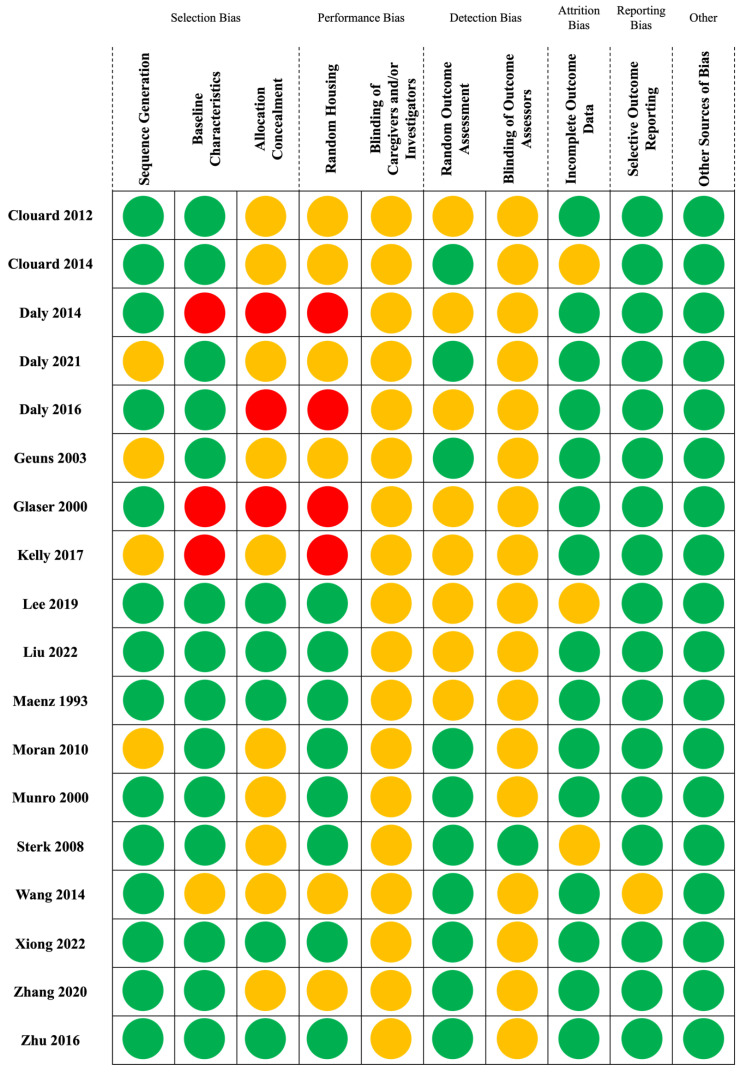
Traffic light plot of risk of bias assessments for included studies. Green = Low risk of bias; Yellow = Unclear; Red = High risk of bias.

The results of risk of bias assessments of the 18 studies are reported in Figure 2. For sequence generation, most studies had a low risk of bias, ensuring proper randomization [30,31,32,33,34,36,38,39,40,41,42,43,44,45]. Regarding baseline characteristics, the majority of studies reported a low risk of bias, indicating comparable baseline characteristics among groups [29,31,32,33,34,36,37,38,39,40,41,42,43,45]. However, allocation concealment had several studies with unclear or high risk, indicating potential biases in the allocation process [22,29,30,32,33,34,36,37,41,42,44,45]. In terms of random housing, there were instances of both low and high risks, reflecting variability in whether housing was randomized. The blinding of caregivers and/or investigators was often marked by an unclear risk due to insufficient reporting on blinding procedures. For random outcome assessment, this domain was often marked by an unclear risk, with few studies explicitly mentioning random outcome assessment [30,31,32,33,34,39,40,41,42,43,44,45]. Similarly, the blinding of outcome assessors had most studies with an unclear risk. Most studies showed a low risk of bias for incomplete outcome data, indicating proper handling of data acquisition and processing [22,29,30,31,34,36,37,39,40,41,42,43,44,45]. Selective outcome reporting was generally low across studies, suggesting comprehensive reporting of outcomes, except for Wang (2014), which was unclear. All studies were rated as low risk for other sources of bias, indicating minimal other sources of bias.

### 3.5. Growth Performance and Feed Preference

#### 3.5.1. Neotame and Neotame + Saccharin

Lee et al. [38] reported that the supplementation of 0.02% neotame significantly increased the average daily gain (ADG) of pigs during the first 7 days of a 14-day feeding period (*p* = 0.049), although this effect was not observed over a 21-day feeding period. Additionally, pigs supplemented with 0.02% neotame had significantly higher average daily feed intake (ADFI) (*p* = 0.047) compared to those supplemented with a 0.02% neotame + saccharin blend during a 14-day period. However, no significant effect on ADFI was seen during week 1 of the 14-day period or throughout a 21-day period. Feed efficiency was not significantly affected by supplementation of 0.02% neotame during the 14- and 21-day feeding periods. Pigs supplemented with a 0.02% neotame + saccharin blend showed a tendency for increased feed efficiency (*p* = 0.055) over the 21-day feeding period, but neither 0.02% nor 0.03% neotame + saccharin blends significantly affected ADG, ADFI, or feed efficiency during the 14- and 21-day periods [38].

Zhu et al. [31] observed that ADG increased quadratically (*p* < 0.05) with increasing dietary neotame levels (10, 20, 30, 40, and 50 mg/kg) during days 1 to 22, 23 to 35, and 1 to 35. ADFI also increased linearly (*p* < 0.05) as neotame levels rose during the same periods and increased quadratically (*p* < 0.05) with increasing neotame levels over the 35-day period. Pigs consuming 30 mg/kg neotame showed significantly higher consumption (*p* < 0.05) compared to a control diet on days 7, 10, and throughout the 15-day feeding period, with a significant increase in feed preference percentage (*p* < 0.05) compared to the control on days 3, 6, 7, 10, and throughout the same period. Zhu et al. [30] also predicted optimal neotame dosages for ADG based on a quadratic plot model: 20.4 and 18.0 mg/kg for days 1 to 22, 22.9 and 22.0 mg/kg for days 23 to 35, and 21.7 and 20.7 mg/kg for the entire 35-day feeding period [31].

#### 3.5.2. Rebaudioside A, Maltodextrin, and Saccharin

Wang et al. [30] demonstrated that rebaudioside A supplementation in 28-day-old weanling pigs led to a linear increase in ADG (*p* ≤ 0.05) and a linear decrease in the feed-to-gain ratio (*p* < 0.05) as the dosage increased from 0 to 300 mg/kg over a 42-day feeding period. The highest ADG was achieved with 300 mg/kg rebaudioside A, making it the most effective dosage among those tested (0, 100, 150, 200, and 250 mg/kg). Additionally, a broken-line regression analysis revealed that the optimal dosage of rebaudioside A to maximize average daily feed intake (ADFI) was 213 mg/kg [30].

Clouard et al. [41] observed that a 2.25% maltodextrin inclusion in water tended to be consumed more (*p* = 0.09) during 12 one-tank training sessions from days 14 to 28 of a 28-day trial period compared to the control diet. Furthermore, the maltodextrin inclusion was consumed significantly more (*p* = 0.008) during these sessions than a 0.37% saccharin inclusion treatment. In contrast, the 0.37% saccharin inclusion in water was consumed significantly less (*p* < 0.05) than both the control diet and the maltodextrin inclusion over the same period [41]. Saccharin supplementation did not significantly affect ADG, feed consumption, or average daily water intake in 28-day-old weanling pigs during a 10-day feeding period [43]. Preference was elicited in pigs when saccharin was supplemented in water at concentrations of 0.2, 0.4, and 0.8 g/L [44].

#### 3.5.3. Stevia

Supplementation of stevioside at levels of 100, 150, 200, 250, and 300 mg/kg linearly increased ADG and ADFI (*p* ≤ 0.05) in 28-day-old pigs during a 42-day feeding period, while also linearly decreased (*p* < 0.05) the feed-to-gain ratio over the same period [30]. Supplementation of 100 to 800 mg/kg had no significant effect on ADG, ADFI, or feed/gain ratio during days 0 to 35 but did linearly increase body weight on day 35 (*p* < 0.05) [40]. The greatest ADG was observed with 300 mg/kg stevioside supplementation during the 42-day feeding period [30] and with 100 mg/kg stevia supplementation during a 75-day feeding period [40]. Supplementation of 83.3, 167, 334 mg/kg [42], 100, 200, 400 mg/kg [39], and 10 to 20% stevia [32] had no significant effect on ADG, ADFI, or feed-to-gain ratio in 24-, 21-, and 26-day-old pigs during 21-, 42-, and 28-day feeding periods, respectively. Pigs supplemented with 167 mg/kg stevia showed the lowest growth during days 0 to 7, and the highest growth during days 8 to 14 of a 21-day feeding period, compared to diets supplemented with 83.3 or 334 mg/kg stevia, and 5% sucrose [42]. Supplementation of 10 to 20% stevia did not significantly affect the initial and final body weight of the pigs but significantly increased feed consumption on day 28 (*p* < 0.01) during a 1-h preference test [32].

#### 3.5.4. Sucralose

Zhang et al. [45] reported that supplementation of 150 mg/kg sucralose significantly increased ADG and ADFI (*p* < 0.05) compared to the supplementation of 75, 225, 300, and 1500 mg/kg sucralose, as well as a control diet, during a 28-day feeding period. However, supplementation at levels of 75, 150, 225, and 300 mg/kg did not significantly affect the gain-to-feed ratio during the same period [45]. A fitted quadratic model revealed that the optimal dosage of sucralose to maximize ADFI was 137.8 mg/kg during days 0 to 14, 145.8 mg/kg during days 15 to 28, and 141.8 mg/kg over the entire 28-day feeding period [45]. Additionally, supplementation with 150 mg/kg sucralose significantly increased feed consumption and preference percentage (*p* < 0.05) compared to the control diet on days 1, 4, and 7, and throughout the entire 28-day feeding period [45]. Preference was also elicited in pigs when sucralose was supplemented in water at concentrations of 0.062 and 0.125 g/L [44].

#### 3.5.5. NHDC + Saccharin

Sterk et al. [33] observed that ADG, ADFI, and the gain-to-feed ratio, as well as the overall time-related development of feed intake and Kaplan-Meier curves for latency time, were not significantly affected by NHDC + saccharin 3D and C-150 supplementation in 26-day-old weanling pigs during a 19-day feeding period. Additionally, supplementation with NHDC + saccharin 3D and C-150 had no significant effect on feed intake characteristics during the first 4 days, days 5 to 11, and days 12 to 19 of the feeding period. However, NHDC + saccharin 3D supplementation significantly increased feed intake (*p* < 0.05) on days 8 and 10, and NHDC + saccharin C-150 tended to increase feed intake (*p* = 0.074) on day 16 compared to the control diet. The average duration of a feeder visit during the first 4 days post-weaning was 23% higher for pigs supplemented with NHDC + saccharin C-150 compared to the control diet. Moreover, the proportion of feeder visits that included feed consumption was significantly higher for NHDC + saccharin C-150 supplemented pigs during days 5 to 11 and days 12 to 19 of the feeding period compared to the control diet [33].

#### 3.5.6. Other Sweeteners

According to two-choice drinking preference tests, alitame (0.15 and 0.3 g/L), acesulfame-K (0.01, 0.05, and 0.35 g/L), and dulcin (0.6, 1.2, and 2.4 g/L) elicited a preference in 2- to 4-month-old pigs [44]. However, monellin, thaumatin, NHDC, P-4000, perillartine, aspartame, and cyclamate did not elicit a preference in the same age group of pigs [44].

### 3.6. Clinical Indicators (Diarrhea, Immune Response, and Biochemical/Oxidative Parameters)

#### 3.6.1. Neotame, Saccharin, and Neotame + Saccharin

Lee et al. [38] reported that a 0.02% neotame + saccharin blend significantly reduced (*p* < 0.05) total blood cholesterol compared to both the control and a 0.03% blend after 14 days. However, the 0.03% blend significantly increased (*p* < 0.05) blood triglycerides compared to the 0.02% blend [38]. Additionally, supplementation with 50 and 500 mg/kg neotame had no significant effect on hematological parameters, serum biochemical parameters, and organ index of pigs throughout a 35-day feeding period [31]. Similarly, supplementation of a saccharin-based sweetener had no significant effect on scours patterns in 28-day old pigs during a 10-day feeding period [43].

#### 3.6.2. Rebaudioside A, NHDC + Saccharin, and Sucralose

Supplementation with 100 to 300 mg/kg rebaudioside A significantly reduced the incidence of diarrhea in 28-day-old pigs during a 42-day feeding period, with a broken line regression analysis identifying 191 mg/kg as the optimal dose to minimize diarrhea incidence [30]. In a 19-day feeding trial, supplementation of NHDC + saccharin 3D and C-150 to 26-day-old weanling pigs had no effect on fecal consistency during the first 5 days postweaning. However, from days 5 to 19, both supplements increased the percentage of firm feces compared to the control diet [33]. In addition, supplementation of 150 and 1500 mg/kg sucralose had no effect on hematological parameters, serum biochemical parameters, or organ index [45].

#### 3.6.3. Stevia

Liu et al. [39] reported that supplementing 100 mg/kg stevia to 21-day-old pigs significantly increased serum catalase (CAT) and liver total superoxide dismutase (T-SOD) activities compared to higher doses and the control diet. Additionally, 100 and 200 mg/kg stevia reduced the rate of diarrhea and malondialdehyde (MDA) content, while higher doses (200 to 800 mg/kg) increased MDA content and enhanced total antioxidant capacity (T-AOC) and glutathione peroxidase (GSH-PX) activity. However, stevia supplementation had no significant effect on serum and liver T-AOC levels, CAT activity, or liver T-SOD at any dosage [39].

Xiong et al. [40] observed that 100 mg/kg stevia significantly reduced serum MDA content compared to higher doses and the control diet. Higher doses (600 to 800 mg/kg) also reduced MDA content, but 800 mg/kg increased triglyceride levels. Stevia supplementation (200 to 800 mg/kg) increased serum T-SOD during both 42- and 75-day feeding periods and resulted in a linear and quadratic increase in serum triglyceride, high-density lipoprotein, albumin, T-SOD, and CAT levels. However, stevia had no significant effect on several other serum markers, including glucose, total protein, cholesterol, and enzyme levels [40].

Wang et al. [30] determined that the optimal stevia dose to reduce diarrhea in 28-day-old pigs during a 42-day feeding period was 251 mg/kg.

### 3.7. Intestinal Development (Taste Receptor/Digestive Enzyme Activity)

#### 3.7.1. Acesulfame K, Aspartame, Cyclamate, Stevia, and Sucralose

Daly et al. [37] reported that supplementing 28-day-old pigs with 10 mM acesulfame K or 2 mM sucralose in drinking water during a 3-day trial significantly increased (*p* < 0.05) the expression and activity of intestinal sodium glucose cotransporter 1 (SGLT1) and activated taste receptors T1R2 and T1R3. In contrast, supplementation with 1 mM aspartame or cyclamate had no effect on T1R2/T1R3 activation or SGLT1 expression and activity [37]. Liu et al. [39] observed that supplementing 100, 200, or 400 mg/kg stevia to 21-day-old pigs over a 42-day feeding period tended to reduce (*p* < 0.05) trypsin, lipase, and amylase activity in the duodenum, but did not significantly affect small intestine morphology or digestive enzyme activity. Similarly, Zhang et al. [45] noted that 150 and 1500 mg/kg sucralose had no significant effect on tissue histopathology compared to a control diet.

#### 3.7.2. Saccharin, NHDC, and NHDC + Saccharin

Moran et al. [34] reported that dietary supplementation with NHDC + saccharin (150 mg/kg body weight) resulted in a 2-fold increase (*p* = 0.001) in SGLT1 mRNA expression and a 1.8-fold increase (*p* = 0.002) in glucose transport. Additionally, supplementation of drinking water with either saccharin (0.25 mM), NHDC (0.02 mM), or a combination of saccharin and NHDC increased SGLT1 mRNA expression in the mid-small intestine by 1.8-fold (*p* = 0.003), 1.6-fold (*p* = 0.016), and 2-fold (*p* = 0.001), respectively. These increases were correlated with rises in SGLT1 protein abundance—1.9-fold (*p* = 0.037), 1.8-fold (*p* = 0.040), and 1.6-fold (*p* = 0.035)—and corresponding increases in glucose uptake. However, no changes in villus height or crypt depth were observed in the intestines following supplementation with NHDC + saccharin or the sweeteners [34].

### 3.8. Gut Microbiota

#### Stevia, Neotame, Saccharin + Neotame, and NHDC + Saccharin

The dietary supplementation of 400, 600, and 800 mg/kg stevia significantly reduced (*p* < 0.05) Chao1 and observed indexes and tended to decrease (*p* = 0.083) the Shannon index during a 75-day feeding period [40]. However, no significant effects on the Chao1, observed-species, Shannon, and Simpson indexes of the colon microbes were observed when 100 to 800 mg/kg stevia was supplemented compared to a control diet [39]. Additionally, no significant differences to the gut microbial structure and Kruckal–Wallis rank sum test results were observed [40]. The supplementation of 400 mg/kg stevia during a 42-day feeding period increased the abundances of the genera Coxiella, Prevotella, Subdoligranulum, Akkermansia, and Roseburia in the intestines of 21-day-old pigs [39]. The supplementation of 400 mg/kg Stevia during a 42-day feeding period also tended to increase the relative abundances of the families Lachnospiraceae (*p* < 0.067) and Coriobacteriaceae (*p* < 0.085) and significantly increased (*p* < 0.05) the relative abundance of the family Prevotellaceae and the genera Roseburia and Prevotella in the colon of 21-day-old pigs compared to a control diet [39]. The dietary supplementation of 0.02% Neotame and a 0.02% saccharin + neotame blend during a 14-day feeding period significantly increased (*p* < 0.05) fecal Lactobacillus abundance compared to a control diet [38].

The dietary supplementation of 0.015% NHDC + saccharin to 28-day-old pigs during a 14-day feeding period significantly increased (*p* < 0.05) abundance of Helicobacteraceae [22], Lactobacullus [35], Lactobacilaceae [36], and lactic acid concentrations [35,36] within the small intestinal mucosa, microbiota, and cecum respectively. However, a significant reduction (*p* < 0.05) in the relative abundance of Campylobacteraceae [22], Veillonellaceae, and Ruminococcaceae [36] in the small intestine and cecum, respectively, was observed. No significant differences were found in the quantitative analysis of total 16s rRNA gene copies in the duodenum and jejunum when 0.015% NHDC + saccharin was supplemented compared to a control diet [22].

### 3.9. Organ Development and Meat Quality

#### Neotame and Stevia

Normal histological structures of the liver and kidney were observed with both 50 and 500 mg/kg Neotame treatments during a 35-day feeding period [31]. The supplementation of 100 mg/kg Stevia significantly increased (*p* < 0.05) hot carcass weight and gastric index and tended to increase (*p* = 0.066) carcass circumference during a 75-day feeding period [40]. As the Stevia supplementation dosage increased from 100 to 800 mg/kg, the score of carcass appearance increased linearly; however, no significant effects were observed on organ index, meat pH and color, drip loss, shear force, marbling score, or the content of intramuscular fat, moisture, myofiber diameter, density of the longissimus thoracis, score of smell, flavor, abnormal flavor, chewiness, juiciness, and turbidness of soup [40]. The supplementation of 1.67 g/kg stevioside for 14 days was completely converted into steriol (853 ± 48 µg/g dry weight) in the feces of treatment pigs; however, no stevioside or steriol was detected in the blood [29].

## 4. Discussion

This systematic review aimed to evaluate the effects of various sweeteners on growth performance, feed intake, and gut health in pigs. The majority of studies focused on growth performance, with several reporting significant improvements in ADG and feed efficiency. Studies on gut health were more limited, but some demonstrated that sweeteners modulated gut microbiota by increasing beneficial bacteria. While the review found consistent evidence supporting growth performance benefits, the limited data on other outcomes, such as immune responses and gut health, preclude definitive conclusions on the broader effects of sweeteners in pig nutrition.

The current findings reveal that supplementation of various sweeteners can exert notable effects on the growth performance and feed preferences of pigs. These effects are influenced by both dosage and the specific type of sweetener used, and several results align with the existing literature while others suggest more complex interactions. Neotame supplementation improved both ADG and ADFI during the early stages of the feeding period [38]. This result is consistent with Zhu et al. [31], who demonstrated a dose-dependent effect of neotame on pig growth rates. These findings suggest that neotame may provide an initial boost in feed intake, possibly by stimulating taste receptors and enhancing palatability. However, neotame’s intense sweetness limits its maximum tolerable dose, as excessive amounts can have negative consequences. Optimal growth rates and feed intake were achieved with diets containing approximately 20 to 30 mg/kg, when different doses up to 50 mg/kg were tested. This is consistent with previous work by Mayhew et al. [46], who reported that rats preferred a basal diet over a diet containing high concentrations (150 mg/kg or more) of neotame. The reduction in feed intake was attributed to the palatability of the diet rather than any toxicological effects. Another possible explanation is that neotame, at high concentrations, might interfere with the natural flavor of the basal diet, leading to reduced consumption, as pigs may avoid the overpowering taste. Interestingly, the negative interaction between neotame and saccharin observed in [38], where the combination led to reduced ADFI compared to neotame alone, raises important questions about the compatibility of sweeteners. Saccharin inclusion in water was consumed significantly less than the control diet [41]. This effect has been noted in other sweeteners like saccharin, where high doses led to decreased feed intake due to similar palatability issues [47]. Moreover, research by Roura and Fu [48] demonstrated that the interaction between taste receptors and sweeteners can affect feed intake, further supporting the idea that compatibility between sweeteners is crucial. Beyond these effects on growth and feed intake, neotame supplementation also appears to influence gut health. A 0.02% neotame or neotame–saccharin blend significantly increased fecal Lactobacillus abundance [38], suggesting potential benefits for gut microbiota. Lactobacillus is associated with improved digestion and nutrient absorption, indicating that neotame may enhance not only palatability but also overall nutrient utilization. These findings suggest a dual role for neotame in improving both feed intake and gut health, though the optimal dosing remains crucial to avoid palatability issues at higher concentrations.

In contrast to neotame, stevioside and rebaudioside A offer more predictable and sustained improvements in growth performance and feed efficiency. Notably, these compounds enhance the growth performance of weaned piglets not only by improving feed palatability but also by potentially reducing diarrhea incidence [30]. The anti-diarrheal effects are thought to be linked to bactericidal properties, particularly against pathogenic bacteria such as Escherichia coli [49]. Therefore, stevioside and rebaudioside A may present a dual benefit in swine production: promoting growth while simultaneously supporting gut health by reducing the risk of infections. In addition to their sweetening properties, both sweeteners have been shown to offer additional therapeutic benefits, including anti-hyperglycemic, anti-hypertensive, anti-inflammatory, anti-diarrheal, and immunomodulatory effects [50]. These multifunctional properties suggest a broader potential for these compounds in animal health management. Supporting evidence from studies in poultry highlights the potential of stevioside as an immunomodulator. For instance, Daneshyar et al. [51] found that 130 mg/kg of stevioside supplementation not only increased the body weight of broiler chickens but also suppressed pro-inflammatory responses following stimulation of the innate immune system. Similarly, Wu et al. [25] reported a linear increase in serum IgG and IgA levels in broilers fed with stevioside, indicating enhanced immune function. These findings imply that stevioside supplementation could be particularly beneficial for young and susceptible animals, such as weaned piglets. Furthermore, Atteh et al. [20] observed that a diet containing 130 mg/kg of stevioside improved body weight and feed conversion ratio in broilers during the first two weeks. Additionally, stevioside altered the short-chain fatty acid profile in the ceca, promoting beneficial microbial changes, such as increases in Bifidobacteria and reductions in Escherichia coli [25]. These microbial changes could further contribute to the observed improvements in growth performance, particularly in young animals. Although there is limited research on the effects of rebaudioside A on gut microbiota in pigs, supplementation of rebaudioside A in mice increased the diversity of fecal Lactobacilli, which is associated with improved gut health [52]. In pigs, increased populations of Lactobacilli have been linked to improved nutrient absorption, better immune function, and overall growth performance [53], making the modulation of gut microbiota a key mechanism through which rebaudioside A positively affects growth. This modulation of the gut microbiota appears to have broader health benefits, particularly in improving gut integrity.

Stevia supplementation has demonstrated clear dose-dependent effects on growth performance, oxidative stress, and gut health in pigs, though its impact can vary based on supplementation duration and animal age. Xiong et al. [40] reported that 100 mg/kg of stevia increased body weight by day 35, although effects on ADG and feed efficiency varied depending on the length of the study. In contrast, other studies [32,42] found no significant impact on ADG or feed efficiency over shorter feeding periods. This variability suggests that stevia’s benefits may become more pronounced with extended supplementation durations, highlighting the importance of study design and animal age when evaluating its effects. In terms of oxidative stress, Liu et al. [39] found that 100 mg/kg of stevia increased antioxidant enzymes such as CAT and SOD, while reducing oxidative stress markers like MDA. Higher doses (200 to 800 mg/kg) further boosted total antioxidant capacity but led to increased serum triglycerides at 800 mg/kg, indicating possible trade-offs at higher levels. These effects were supported by research in rats, which showed that stevia supplementation offers protective benefits against diseases such as ulcerative colitis, hyperuricemia, diabetes mellitus, and acute liver injury, primarily due to its antioxidant properties [54]. Stevia’s antioxidant effects are attributed to its high polyphenol content, including phenolic acids and flavonoids, which neutralize reactive oxygen species (ROS) by stabilizing them and preventing cellular damage. Additionally, stevia enhances the activity of key antioxidant enzymes like SOD, CAT, and glutathione peroxidase (GPx), further reducing oxidative stress and inflammation. This dual mechanism supports better cellular health and may help mitigate oxidative damage in animals. Stevia supplementation also had notable effects on gut microbial composition, although its impact on microbial diversity was mixed. Xiong et al. [40] found that higher doses of stevia (400, 600, and 800 mg/kg) reduced microbial diversity during a 75-day feeding period, while Liu et al. [39] observed no significant changes in diversity across various doses. However, stevia at 400 mg/kg positively influenced the abundance of beneficial gut microbes, including Lachnospiraceae, Coriobacteriaceae, and Prevotellaceae, suggesting that moderate doses of stevia can enhance the gut microbial profile even if overall diversity remains unaffected. Similar effects were observed in recent poultry studies, where stevia supplementation modulated intestinal microbial composition and improved production performance, egg nutrition, gut health, and immune capabilities in laying hens [55,56]. In broilers, stevia enhanced intestinal functionality, increased microbial diversity, and promoted the growth of beneficial bacterial genera [57]. These findings suggest that stevia’s role in supporting gut health and microbial balance may extend across species, further highlighting its potential as a dietary supplement for improving both animal performance and gut integrity.

Sucralose and NHDC + saccharin have shown potential to influence growth performance, though their effects vary depending on the dose. Sucralose supplementation at 150 mg/kg significantly improved ADG and ADFI [45], suggesting that moderate doses can enhance feed intake and growth in pigs. However, the lack of significant changes in the gain-to-feed ratio indicates that, while sucralose may boost intake, it does not necessarily improve feed efficiency. In contrast, NHDC + saccharin showed less consistent results. Sterk et al. [33] found no significant impact on ADG or ADFI during a 19-day period, although NHDC + saccharin increased feed intake on certain days and extended feeder visits. This suggests that NHDC + saccharin may improve feeding behavior without directly influencing overall growth performance. Similar trends were observed in ruminants, where NHDC + saccharin supplementation showed only a tendency to increase feed intake [15] and exhibited a slight tendency to increase ADG during a 56-day receiving period [19]. The improvements in feed intake seen with both sucralose and NHDC + saccharin suggest that these sweeteners can enhance palatability, which is particularly beneficial for weaning pigs, a period when appetite is often reduced. However, the inconsistent effects on growth efficiency raise questions about their long-term value in improving overall feed conversion. Sucralose and NHDC + saccharin have demonstrated benefits in nutrient absorption and gut health, primarily by enhancing the activity of the SGLT1 and activating taste receptors, potentially improving glucose absorption and energy utilization in pigs [34,37]. However, neither sweetener significantly affected intestinal morphology, indicating their role is focused on nutrient transport rather than structural development. NHDC + saccharin showed a more pronounced effect on gut microbiota than sucralose, increasing the abundance of beneficial bacteria such as Lactobacillus and reducing harmful populations like Campylobacteraceae [22]. This suggests that NHDC + saccharin may improve gut health and immune function, potentially leading to better overall performance. In contrast, the effect of sucralose on gut microbiota remains unclear, with limited data available, indicating the need for further research. A comprehensive review suggests short-term changes in the microbiota with sucralose consumption [58], though more long-term research is needed to fully understand its effects. For instance, inconsistent findings have shown that sucralose reduced obesity in humans by decreasing the Firmicutes/Bacteroidetes ratio and increasing Actinobacteria. However, in mice and rats, sucralose was found to induce obesity by also reducing the Firmicutes/Bacteroidetes ratio [59]. These conflicting results highlight the need for further studies to clarify sucralose’s impact on gut health across different species and durations of use. Clinically, sucralose showed no adverse effects on health markers [45], while NHDC + saccharin improved post-weaning fecal firmness, indicating added gut health benefits during the transition period for young pigs [33]. Overall, both sweeteners are safe at tested doses, with NHDC + saccharin offering additional potential for enhancing gut health by positively modulating microbial populations and supporting immune function.

## 5. Strengths and Limitations

The strengths of this systematic review lie in its comprehensive evaluation of the effects of various sweeteners on growth performance, feed intake, and gut health in pigs. The review effectively highlights consistent improvements in ADG and feed efficiency, demonstrating the potential of sweeteners to enhance pig growth, particularly during critical periods like weaning when appetite is often reduced. Additionally, it emphasizes the benefits of sweeteners in modulating gut microbiota, potentially improving nutrient absorption and supporting immune function. However, several limitations were identified. There is a lack of research on the effects of combining different sweeteners, where interactions may reduce feed intake or alter other outcomes, raising concerns about compatibility and the need for further investigation. Furthermore, there are limited data on the long-term effects of sweeteners, particularly regarding gut health and immune responses, making it difficult to form comprehensive conclusions. The variability in effects on feed efficiency and gut microbiota across studies also suggests a need for more exploration of underlying pathways. While the review discusses potential mechanisms, such as the activation of taste receptors and modulation of gut bacteria, more detailed mechanistic studies are required to fully understand how sweeteners influence growth, metabolism, and health outcomes in pigs.

## 6. Conclusions

This systematic review highlights the potential of various sweeteners to improve growth performance, feed intake, and gut health in pigs. Many studies reported significant improvements in ADG and feed efficiency, suggesting that sweeteners can be effective in promoting pig growth, particularly during critical periods such as weaning when appetite is reduced. Additionally, certain sweeteners demonstrated the ability to modulate gut microbiota by increasing beneficial bacteria, potentially enhancing nutrient absorption and supporting immune function. However, despite the promising results, limitations were identified. Research on gut health and immune responses remains limited, and the long-term effects of sweetener use in pig diets require further investigation. Additionally, the review raises concerns about the interactions between different sweeteners, as some combinations resulted in reduced feed intake. This highlights the need for more studies exploring the compatibility and mechanisms of action of sweeteners. Furthermore, the variability in findings across studies suggests that dosage, type of sweetener, and study design play crucial roles in determining their effectiveness. Overall, while sweeteners show potential in enhancing pig nutrition, further research is needed to fully understand their broader impacts and optimize their use in swine production systems.

## Figures and Tables

**Table 1:** Summary of inclusion and exclusion criteria.

	Inclusion Criteria	Exclusion Criteria
Population (P)	Pigs (*Sus scrofa* and/or *sus domesticus*) Breeds: Commonly industrial used	Non-pig species (e.g., guinea pig)
Intervention (I)	Dietary supplementation with various non-nutritive sweeteners	Non-nutritive sweeteners are combined with the treatment
Comparators (C)	Control groups fed a basal or commercial diet without non-nutritive sweeteners Additional comparators: Different concentrations/types of non-nutritive sweeteners and combinations in both feed and water	Studies without a control group
Outcomes (O)	Growth performance Incidence of diarrhea and overall health status Feed palatability and preference Gut health and microbiota composition Intestinal development Blood biochemical parameters	Studies only measured in vitro or ex vivo
Study designs (S)	Controlled experimental trials with random allocation to treatment and control groups	Observational studies, including cross-sectional, cohort, and case-control studies Studies without a clear intervention, detailed methodology, or outcomes measurement procedures

**Table 2:** Search strategies for PubMed, Scopus, Web of Science, and Agricola.

PubMed	
Query	((“non-nutritive sweeteners”[All Fields] OR “artificial sweeteners”[All Fields] OR “sugar substitutes”[All Fields] OR “sweetener”[All Fields] OR “sweeteners”[All Fields] OR “Sweetening Agents”[MeSH Terms]) AND (“Piglet”[All Fields] OR “Piglets”[All Fields] OR “Pig”[All Fields] OR “Pigs”[All Fields] OR “Swine”[All Fields] OR “Porcine”[All Fields] OR “Sus scrofa”[All Fields])) AND (1990/1/1:2024/12/12[pdat])
Language	Limited by English
Range	Year 1990–2024
Scopus	
Query	(“non-nutritive sweeteners” OR “artificial sweeteners” OR “sugar substitutes” OR “sweetener” OR “sweeteners” OR “Sweetening Agents”) AND (“Piglet” OR “Piglets” OR “Pig” OR “Pigs” OR “Swine” OR “Porcine” OR “Sus scrofa”) AND PUBYEAR > 1989 AND PUBYEAR < 2026 AND (LIMIT-TO (EXACTKEYWORD, “Swine”) OR LIMIT-TO (EXACTKEYWORD, “Pig”)) AND (LIMIT-TO (LANGUAGE, “English”))
Language	Limited by English
Range	Year 1990–2024
Web of Science	
Query	TS = (“non-nutritive sweeteners” OR “artificial sweeteners” OR “sugar substitutes” sweetener OR “Sweetening Agents” OR sweeteners) AND TS = (pig OR pigs OR piglet OR piglets OR swine OR porcine or “Sus scrofa”) | Timespan: 1 January 1990 to 31 December 2024 (Publication Date)
Language	Limited by English
Range	Year 1990–2024
Agricola	
Query	“non-nutritive sweeteners” OR “non-nutritive sweetener” OR “artificial sweeteners” OR “artificial sweetener” OR “sugar substitutes” OR “sugar substitute “OR “sweetener” OR “sweeteners” OR “Sweetening Agents” AND “Piglet” OR “Piglets” OR “Pig” OR “Pigs” OR “Swine” OR “Porcine” OR “Sus scrofa”
Language	Limited by English
Range	Year 1990–2024

**Table 3:** The summary of study and main results of non-nutritive sweeteners in pigs ^1^.

Authors, Year, Country	Number of Animals/Species	Age	Type of Sweetener/Number Per Group	Experiment Duration	Characteristics Evaluated	Main Results
Kelly et al. (2017), UK [22]	16, Gloucestershire Old Spot pigs	28 days	0.015% NHDC + Saccharin	2 weeks	Mucosa-associated microbiota	Relative abundances of 76 small intestinal and 81 large intestinal mucosa associated OTU’s were different among the NHDC + Saccharin treatment, respectively Relative abundance levels of total *Campylobacteraceae* were lower throughout the small intestine, and large intestine, with NHDC + Saccharin treatment *Helicobacteraceae* abundance increased within the small intestine of the NHDC + Saccharintreated group A *Lactobacillus gasseri* related OTU abundance was increased in the large intestine of NHDC + Saccharin-treated group
Geuns et al. (2003), Belgium [29]	12 female pigs	Not specified; initial body weight: approximately 23 kg	1.67 g/kg stevioside (n = 6)	14 day	Metabolism of stevioside to steviol in the feces Absorption of stevioside and steviol in the blood	Stevioside was completely converted into steviol in feces Steviol was noted to have concentrations around 853 ± 48 µg/g dry weight in the feces No stevioside or steviol was detected in the blood
Wang et al. (2014), China [30]	*Experiment 1 and 2:* 216. Duroc × Landrace × Large White pigs, respectively	28 ± 1 days, respectively	*Experiment 1:* 100, 150, 200, 250, or 300 mg/kg stevioside (n = 36, respectively) *Experiment 2:* 100, 150, 200, 250, or 300 mg/kg rebaudioside A (n = 36, respectively)	42 day, respectively	Growth performance Diarrhea incidence rate	*Experiment 1:* Stevioside increased ADG linearly throughout the experiment ADFI increased linearly with higher doses of stevioside 251 mg/kg stevioside showed lowest incidence of diarrhea based on the broken-line regression model Optimal dosage for dietary stevioside supplementation: 200 to 250 mg/kg *Experiment 2:* Rebaudioside A increased ADG linearly throughout the experiment Feed:gain decreased linearly as the mg/kg of rebaudioside A treatment increased 213 mg/kg rebaudioside A showed greatest ADFI according to the broken-1 line regression model The lowest diarrhea incidence was observed at a dose of 191 mg/kg based on the broken-line regression analysis
Zhu et al. (2016), China [31]	*Experiment 1:* 48, Duroc × Landrace × Large White pigs *Experiment 2:* 216, Duroc × Landrace × Large White pigs *Experiment 3:* 108, Duroc × Landrace × Large White pigs	*Experiment 1:* Not specified; initial body weight: 9.05 ± 0.04 kg *Experiment 2:* Not specified; initial body weight: 7.35 ± 0.06 kg *Experiment 3:* Not specified; initial body weight: 7.34 ± 0.08 kg	*Experiment 1:* 30 mg/kg neotame *Experiment 2:* 10, 20, 30, 40, or 50 mg/kg neotame *Experiment 3:* 50 or 500 mg/kg neotame	*Experiment 1:* 15 days *Experiment 2:* 35 days *Experiment 3:* 35 days	*Experiment 1:* Feed intake Diet preference percentage *Experiment 2:* Growth performance *Experiment 3:* Hematological parameters Serum biochemical parameters Histopathological parameters	*Experiment 1:* 30 mg/kg neotame significantly increased feed intake during day 7 and 10, and 1 to 10 30 mg/kg neotame increased diet preference percentage during day 3, 6, 7, 10, and 1 to 10 *Experiment 2:* A linear increase in ADFI was observed when increasing neotame levels during day 1 to 22 and 1 to 35 ADG and ADFI increased quadratically during day 1 to 22, 23 to 35, and 1 to 35 when neotame levels increased Optimal concentrations for maximum ADG and ADFI during the entire experimental period were 21.7 mg/kg and 20.7 mg/kg, respectively *Experiment 3:* No adverse effects on hematological or serum biochemical parameters were observed Normal histological structures were present in the liver and kidney at up to 500 mg/kg neotame
Clouard and Val-Laillet (2014), France [32]	32 female, Large White/Landrace × Pietrain pigs	26.38 ± 0.09 days	10 to 20% stevia rebaudiana, minimum of 90% steviol glycosides, 5 to 10% high-saponin plants extract, and 70 to 85% excipients (n = 8, respectively)	28 days	Growth performance Feed preference	No significant effects of feed additives on growth performance Feed consumption of 10 to 20% stevia rebaudiana was increased on day 23 (1-h preference test)
Sterk et al. (2008), The Netherlands [33]	198, Great Yorkshire × Dutch Landrace × D-line pigs	26 days	150 mg/kg of NHDC + Saccharin C-150 (n = 66) 150 mg/kg of NHDC + Saccharin 3D (n = 66)	19 days	Growth performance Feed intake per visit (g) Latency time, total visits per day Number of visits with food consumed Fecal consistency	No significant differences between day 0 to 11 were observed between feed intake characteristics 3D treatment had an increased percentage of visits with feed intake between day 12 to 19 and increased feed intake on day 8 and 10 Sweetener diets decreased the percentage of soft feces
Moran et al. (2010), UK [34]	56, Landrace × Large White pigs	28 days	150 mg/kg body weight NHDC + Saccharin in feed (n = 12) 150 mg/kg body weight NHDC + Saccharin in water (n = 8) 34.8 mg/d/animal saccharin in water (n = 8) 8.7 mg/d/animal NHDC in water (n = 8) NHDC + Saccharin + inhibitor of human sweet taste receptor in water (n = 4)	3 days	Expression of Na +/glucose co-transporter (SGLT1) Detection of gut hormones and sweet taste receptors	Expression of SGLT1 mRNA and relative protein abundance was increased by sweetener treatments D-Glucose uptake was increased by sweetener treatments Sweet taste receptor T1R2/T1R3 and gustducin were co-expressed in the intestine when supplemented with sweeteners
Daly et al. (2014), UK [35]	24, Landrace × Large White pigs	28 days	5% lactose 0.015% NHDC + Saccharin	2 weeks	Gut microbiota Cecal lactic acid concentration	NHDC + Saccharin increased populations of *Lactobacillus* in the cecal microbiota NHDC + Saccharin increased cecal lactic acid concentration
Daly et al. (2016), UK [36]	16, Landrace × Large White pigs	28 days	0.015% NHDC + Saccharin	2 weeks	Gut microbiota Cecal short chain fatty acid concentration analysis	NHDC + Saccharin increased *Lactobacillaceae* and reduced *Veillonellaceae* and *Ruminococcaaceae* abundance in the cecum NHDC + Saccharin increased cecal lactic acid concentration NHDC + Saccharin enhanced the expression of *Lactobacillus* 4228 (sugar transporters) in vitro
Daly et al. (2021), UK [37]	24, Landrace × Large White pigs	28 days	2 mM sucralose 0.25 mM saccharin 10 mM acesulfame-K 1 mM aspartame 10 mM cyclamate (n = 4, respectively)	3 days	Activation of pig sweet taste receptor T1R2-T1R3 Intestinal capacity of glucose uptake	Sucralose, saccharin, and acesulfame K increased T1R2-T1R3 receptor activation Sucralose, saccharin, and acesulfame K resulted in increased expression and activity of intestinal SGLT1, enhancing glucose absorption.
Lee et al. (2019), South Korea [38]	*Experiment 1:* 30, Landrace × Yorkshire × Duroc pigs *Experiment 2:* 52 pigs.	*Experiment 1:* Not specified; initial body weight: 7.01 ± 0.3 kg *Experiment 2:* Not specified; initial body weight: 18.67 ± 0.52 kg	*Experiment 1:* 0.05% saccharin (50% saccharin-natrium) 0.03% saccharin-neotame (50% saccharin-natrium + 2% neotame) 0.02% neotame (10% neotame) 0.02% saccharin-neotame mix (10% saccharin-natrium + 10% neotame) *Experiment 2:* 0.05% saccharin (50% saccharin-natrium) 0.03% saccharin-neotame mix (50% saccharin-natrium + 2% neotame) 0.02% neotame (10% neotame) 0.02% saccharin-neotame mix (10% saccharin-natrium + 10% neotame)	*Experiment 1:* 21 day *Experiment 2:* 14 day	*Experiment 1:* Growth performance Feed preference *Experiment 2:* Growth performance Nutrient digestibility Blood biochemical analysis Fecal bacterial counts	*Experiment 1:* 0.02% Saccharin-neotame mix treatment had a high tendency to increase gain:feed throughout day 0 to 21 0.02% Neotame treatment increased feed preference during day 0 to 21 *Experiment 2:* ADG was highest in 0.02% saccharin-neotame mix treatment during the first week ADFI was highest in 0.02% saccharin-neotame mix treatment throughout the experiment Fecal *Lactobacillus* counts were higher in the 0.02% saccharin-neotame mix reduced blood cholesterol, while 0.02% neotame groups
Liu et al. (2022), China [39]	108, Duroc × Landrace × Yorkshire pigs	21 days	100, 200, or 400 mg/kg stevia residue extract (n = 36, respectively)	42 days	Growth performance Diarrhea rate Antioxidant capacity Intestinal health Gut microbiota	100 and 200 mg/kg stevia residue extract decreased the rate of diarrhea during day 29 to 42 200 mg/kg stevia residue extract reduced the rate of diarrhea throughout the experiment Stevia residue extract reduced malondialdehyde content and increased total superoxide dismutase in serum and liver 200 and 400 mg/kg stevia residue extract increased total antioxidant capacity Relative abundance of *Prevotellaceae* (Family) and *Roseburia* and *Prevotella* (Genus) increased with 400 mg/kg stevia residue extract
Xiong et al. (2022), China [40]	48, Duroc × Landrace × Yorkshire pigs	Not specified; initial body weight: 68.08 ± 0.74 kg	100, 200, 400, 600, or 800 mg/kg stevia residue extract (n = 8, respectively)	75 days	Growth performance Carcass traits Meat quality Antioxidant capacity Gut microbiota	Stevia residue extract supplementation linearly increased body weight on day 35 Stevia residue extract supplementation increased ADG and tended to increase ADFI from day 1 to 75, with the highest gain observed at 100 mg/kg Hot carcass weight, and gastric index increased, and tended to increase circumference with the supplementation of 100 mg/kg stevia residue extract. Triglyceride content within serum was increased in the 800 mg/kg stevia residue extract treatment Malondialdehyde content was lower in 100 mg/kg Stevia residue extract compared to other treatments 600 and 800 mg/kg stevia residue extract reduced malondialdehyde content within the *longissimus thoracis* compared to the control Stevia residues extract increased serum total superoxide dismutase activity Complexity of species diversity of 400, 600, and 800 mg/kg was decreased within Chao1 and observed indexes and tended to decrease the Shannon index
Clouard et al. (2012), France [41]	27, Large White/Landrace × Large White pigs	Not specified; initial body weight: 34.1 ± 2.5 kg	2.25% maltodextrin (n = 3/experiment) 0.37% saccharin (n = 3/experiment)	1 week habituation + 2 weeks conditioning sessions	Feed and water consumption Two-choice drinking and feeding test	Maltodextrin supplementation increased water consumption during the conditioning sessions
Munro et al. (2000), Canada [42]	209, Purebred Yorkshire pigs	24 ± 4 days	5% sucrose 83.3 mg/kg stevia 167 mg/kg stevia 334 mg/kg stevia	3 weeks	Growth performance	167 mg/kg stevia reduced body weight during week 1 post-weaning 5% sucrose increased feed intake and feed:gain throughout the trial
Maenz et al. (1993), Canada [43]	12 pigs/trial with 4 replicate trials.	28 days	Palasweet (saccharin-based sweetener)	10 days	Growth performance Water intake Diarrhea score	No significant effects of sweetener were observed
Glaser et al. (2000), Switzerland [44]	75 pigs	2 to 4 months	0.01, 0.05, or 0.35 g/L acesulfame-K (n = 4, respectively) 0.15 or 0.3 g/L alitame (n = 4, respectively) 1.5, 3, or 5 g/L aspartame (n = 4, respectively) 5, 10, or 20 g/L cyclamate (n = 4, respectively) 0.6, 1.2, 2.4 g/L dulcin (n = 4, respectively) 0.2 g/L monellin (n = 4) 0.6 g/L NHDC (n = 4) 0.05 g/L 5-Nitro-2-propoxy aniline (P-4000) (n = 4) 2.5 g/L perillartine (n = 4, respectively) 0.2, 0.4, or 0.8 g/L saccharin (n = 4, respectively) 0.062 or 0.125 g/L sucralose (n = 5 to 6, respectively) 0.20 g/L Thaumatin (n = 4)		Gustatory responses Preference test Richter-type drinking test	Monellin, thaumatin, NHDC, P-4000, perillartine, aspartame, and cyclamate did not elicit preference in pigs Alitame, sucralose, saccharin, acesulfame-K, and dulcin elicit appeal in pigs
Zhang et al. (2020), China [45]	*Experiment 1:* 48 pigs *Experiment 2:* 180 pigs *Experiment 3:* 108 pigs	*Experiment 1:* Not specified; initial body weight: 8.90 ± 0.09 kg *Experiment 2:* Not specified; initial body weight: 7.95 ± 0.17 kg *Experiment 3:* Not specified; initial body weight: 7.97 ± 0.18 kg	*Experiment 1:* 150 mg/kg sucralose (n = 48) *Experiment 2:* 75, 150, 225, or 300 mg/kg sucralose (n = 36, respectively) *Experiment 3:* 150 or 1500 mg/kg sucralose (n = 36, respectively)	*Experiment 1:* 15 days *Experiment 2:* 28 days *Experiment 3:* 28 days	*Experiment 1:* Feed intake Diet preference percentage *Experiment 2:* Growth performance *Experiment 3:* Growth performance Hematological parameters Serum Parameters Organ index Histopathological analysis	*Experiment 1:* Higher diet preference percentage for sucralose treatment during the entire experimental period *Experiment 2:* 150 mg/kg sucralose increased ADG throughout day 15 to 28, and the entire experimental period (day 0 to 28) 150 mg/kg sucralose increased ADFI during day 0 to 14, 15 to 28, and 0 to 28 225 and 300 mg/kg sucralose decreased ADFI throughout the experiment Maximum level of inclusion to maximize ADG was 146.7 mg/kg, 150.l mg/kg, and 149.6 mg/kg of sucralose for day 0 to 14, 15 to 28, and 0 to 28, respectively Maximum level of inclusion to maximize ADFI was 137.8 mg/kg, 145.8 mg/kg, and 141.8 mg/kg of sucralose for day 0 to 14, 15 to 28, and 0 to 28, respectively *Experiment 3:* 150 mg/kg sucralose had higher ADG and ADFI than control and 1500 mg/kg group throughout the experiment No significant differences of hematological, serum parameters, histopathological analysis, and organ index were noted among treatments

^1^ ADG = Average daily gain; ADFI = Average daily feed intake; NHDC = Neohesperidin dihydrochalcone; SGLT 1 = Sodium glucose cotransporter 1; NHDC + Saccharin = A combination of NHDC and saccharin.

## References

[1] Carocho M., Morales P., Ferreira I.C.F.R. (2017). Sweeteners as Food Additives in the XXI Century: A Review of What Is Known, and What Is to Come. Food Chem. Toxicol..

[2] Chen J., Lei Y., Zhang Y., He S., Liu L., Dong X. (2020). Beyond Sweetness: The High-Intensity Sweeteners and Farm Animals. Anim. Feed Sci. Technol..

[3] Conz A., Salmona M., Diomede L. (2023). Effect of Non-Nutritive Sweeteners on the Gut Microbiota. Nutrients.

[4] Walters D.E., Cyclamate H.P.S. (2013). The Sweetener Book.

[5] Yalamanchi S., Srinath R., Dobs A., Caballero B., Finglas P.M., Toldrá F. (2016). Acesulfame-K. Encyclopedia of Food and Health.

[6] Basson A.R., Rodriguez-Palacios A., Cominelli F. (2021). Artificial Sweeteners: History and New Concepts on Inflammation. Front. Nutr..

[7] European Parlament Official Journal of the European Union L346/2006. https://eur-lex.europa.eu/legal-content/EN/TXT/HTML/?uri=OJ%3AL%3A2006%3A346%3AFULL.

[8] Food and Drug Administation HHS Title 21. https://www.ecfr.gov/current/title-21.

[9] Aldinger S.M., Speer V.C., Hays V.W., Catron D.V. (1959). Effect of Saccharin on Consumption of Starter Rations by Baby Pigs. J. Anim. Sci..

[10] Goatcher W.D., Church D.C. (1970). Taste Responses in Ruminants. I. Reactions of Sheep to Sugars, Saccharin, Ethanol and Salts. J. Anim. Sci..

[11] Hellekant G., Hård af Segerstad C., Roberts T.W. (1994). Sweet Taste in the Calf: III. Behavioral Responses to Sweeteners. Physiol. Behav..

[12] Moran A.W., Al-Rammahi M., Zhang C., Bravo D., Calsamiglia S., Shirazi-Beechey S.P. (2014). Sweet Taste Receptor Expression in Ruminant Intestine and Its Activation by Artificial Sweeteners to Regulate Glucose Absorption. J. Dairy Sci..

[13] Koester L.R., Anderson C.J., Cortes B.W., Lyte M., Schmitz-Esser S. (2020). Influence of the Artificial Sodium Saccharin Sweetener Sucram^®^ on the Microbial Community Composition in the Rumen Content and Attached to the Rumen Epithelium in Dairy Cattle: A Pilot Study. BioRxiv.

[14] Koester L.R., Hayman K., Anderson C.J., Tibbs-Cortes B.W., Daniels K.M., Seggerman F.M., Gorden P.J., Lyte M., Schmitz-Esser S. (2022). Influence of a Sodium-Saccharin Sweetener on the Rumen Content and Rumen Epithelium Microbiota in Dairy Cattle during Heat Stress. J. Anim. Sci..

[15] Ponce C.H., Brown M.S., Silva J.S., Schlegel P., Rounds W., Hallford D.M. (2014). Effects of a Dietary Sweetener on Growth Performance and Health of Stressed Beef Calves and on Diet Digestibility and Plasma and Urinary Metabolite Concentrations of Healthy Calves. J. Anim. Sci..

[16] Siurana A., Wall E., Rodrguez M., Castillejos L., Ferret A., Calsamiglia S. (2014). The Effect of Dietary Supplementation of Artificial Sweetener on Performance of Milk-Fed Calves. J. Anim. Sci..

[17] Jiang J., Liu S., Jamal T., Ding T., Qi L., Lv Z., Yu D., Shi F. (2020). Effects of Dietary Sweeteners Supplementation on Growth Performance, Serum Biochemicals, and Jejunal Physiological Functions of Broiler Chickens. Poult. Sci..

[18] Molina-Barrios R.M., Avilés-Trejo C.R., Puentes-Mercado M.E., Cedillo-Cobián J.R., Hernández-Chavez J.F. (2021). Effect of Dietary Stevia-Based Sweetener on Body Weight and Humoral Immune Response of Broiler Chickens. Vet. World.

[19] McMeniman J.P., Rivera J.D., Schlegel P., Rounds W., Galyean M.L. (2006). Effects of an Artificial Sweetener on Health, Performance, and Dietary Preference of Feedlot Cattle. J. Anim. Sci..

[20] Atteh J.O., Onagbesan O.M., Tona K., Decuypere E., Geuns J.M.C., Buyse J. (2008). Evaluation of Supplementary Stevia (*Stevia rebaudiana*, *Bertoni*) Leaves and Stevioside in Broiler Diets: Effects on Feed Intake, Nutrient Metabolism, Blood Parameters and Growth Performance. J. Anim. Physiol. Anim. Nutr..

[21] Sigalet D.L., Wallace L., De Heuval E., Sharkey K.A. (2010). The Effects of Glucagon-like Peptide 2 on Enteric Neurons in Intestinal Inflammation. Neurogastroenterol. Motil..

[22] Kelly J., Daly K., Moran A.W., Ryan S., Bravo D., Shirazi-Beechey S.P. (2017). Composition and Diversity of Mucosa-associated Microbiota along the Entire Length of the Pig Gastrointestinal Tract; Dietary Influences. Environ. Microbiol..

[23] Connor E.E., Evock-Clover C.M., Wall E.H., Baldwin R.L., Santin-Duran M., Elsasser T.H., Bravo D.M. (2016). Glucagon-like Peptide 2 and Its Beneficial Effects on Gut Function and Health in Production Animals. Domest. Anim. Endocrinol..

[24] Sun D., Liu L., Mao S., Zhu W., Liu J. (2019). Aspartame Supplementation in Starter Accelerates Small Intestinal Epithelial Cell Cycle and Stimulates Secretion of Glucagon-like Peptide-2 in Pre-Weaned Lambs. J. Anim. Physiol. Anim. Nutr..

[25] Wu X., Yang P., Sifa D., Wen Z. (2019). Effect of Dietary Stevioside Supplementation on Growth Performance, Nutrient Digestibility, Serum Parameters, and Intestinal Microflora in Broilers. Food Funct..

[26] Kim K., Jansen M. Evaluating the Effects of Non-Nutritive Sweeteners on Pigs: A Systematic Review. PROSPERO, 2024. https://www.crd.york.ac.uk/prospero/display_record.php?ID=CRD42024518080.

[27] Schardt C., Adams M.B., Owens T., Keitz S., Fontelo P. (2007). Utilization of the PICO Framework to Improve Searching PubMed for Clinical Questions. BMC Med. Inform. Decis. Mak..

[28] Covidence Systematic Review Software, Veritas Health Innovation, Melbourne, Australia, 2024. www.covidence.org.

[29] Geuns J.M.C., Augustijns P., Mols R., Buyse J.G., Driessen B. (2003). Metabolism of Stevioside in Pigs and Intestinal Absorption Characteristics of Stevioside, Rebaudioside A and Steviol. Food Chem. Toxicol..

[30] Wang L.S., Shi Z., Shi B.M., Shan A.S. (2014). Effects of Dietary Stevioside/Rebaudioside A on the Growth Performance and Diarrhea Incidence of Weaned Piglets. Anim. Feed Sci. Technol..

[31] Zhu L., Wang G., Dong B., Peng C.C., Tian Y.Y., Gong L.M. (2016). Effects of Sweetener Neotame on Diet Preference, Performance and Hematological and Biochemical Parameters of Weaned Piglets. Anim. Feed Sci. Technol..

[32] Clouard C., Val-Laillet D. (2014). Impact of Sensory Feed Additives on Feed Intake, Feed Preferences, and Growth of Female Piglets during the Early Postweaning Period1. J. Anim. Sci..

[33] Sterk A., Schlegel P., Mul A.J., Ubbink-Blanksma M., Bruininx E.M.A.M. (2008). Effects of Sweeteners on Individual Feed Intake Characteristics and Performance in Group-Housed Weanling Pigs1. J. Anim. Sci..

[34] Moran A.W., Al-Rammahi M.A., Arora D.K., Batchelor D.J., Coulter E.A., Daly K., Ionescu C., Bravo D., Shirazi-Beechey S.P. (2010). Expression of Na^+^/Glucose Co-Transporter 1 (SGLT1) Is Enhanced by Supplementation of the Diet of Weaning Piglets with Artificial Sweeteners. Br. J. Nutr..

[35] Daly K., Darby A.C., Hall N., Nau A., Bravo D., Shirazi-Beechey S.P. (2014). Dietary Supplementation with Lactose or Artificial Sweetener Enhances Swine Gut *Lactobacillus* Population Abundance. Br. J. Nutr..

[36] Daly K., Darby A.C., Hall N., Wilkinson M.C., Pongchaikul P., Bravo D., Shirazi-Beechey S.P. (2016). Bacterial Sensing Underlies Artificial Sweetener-induced Growth of Gut *Lactobacillus*. Environ. Microbiol..

[37] Daly K., Moran A.W., Al-Rammahi M., Weatherburn D., Shirazi-Beechey S.P. (2021). Non-Nutritive Sweetener Activation of the Pig Sweet Taste Receptor T1R2-T1R3 In Vitro Mirrors Sweetener Stimulation of the Gut-Expressed Receptor In Vivo. Biochem. Biophys. Res. Commun..

[38] Lee C.H., Yun W., Lee J.H., Kwak W.G., Oh H.J., An J.S., Liu S.D., Cho J.H. (2019). Effects of Artificial Sweeteners on Feed Palatability and Performance in Weaned Pigs. Can. J. Anim. Sci..

[39] Liu S., Xiong Y., Cao S., Wen X., Xiao H., Li Y., Chi L., He D., Jiang Z., Wang L. (2022). Dietary Stevia Residue Extract Supplementation Improves Antioxidant Capacity and Intestinal Microbial Composition of Weaned Piglets. Antioxidants.

[40] Xiong Y., Liu S., Xiao H., Wu Q., Chi L., Zhu L., Fang L., Li Y., Jiang Z., Wang L. (2022). Dietary Stevia Residue Extract Supplementation Improves the Performance and Antioxidative Capacity of Growing–Finishing Pigs. J. Sci. Food Agric..

[41] Clouard C., Chataignier M., Meunier-Salaün M.-C., Val-Laillet D. (2012). Flavour Preference Acquired via a Beverage-Induced Conditioning and Its Transposition to Solid Food: Sucrose but Not Maltodextrin or Saccharin Induced Significant Flavour Preferences in Pigs. Appl. Anim. Behav. Sci..

[42] Munro P.J., Lirette A., Anderson D.M., Ju H.Y. (2000). Effects of a New Sweetener, Stevia, on Performance of Newly Weaned Pigs. Can. J. Anim. Sci..

[43] Maenz D.D., Patience J.F., Wolynetz M.S. (1993). Effect of Water Sweetener on the Performance of Newly Weaned Pigs Offered Medicated and Unmedicated Feed. Can. J. Anim. Sci..

[44] Glaser D., Wanner M., Tinti J.M., Nofre C. (2000). Gustatory Responses of Pigs to Various Natural and Artificial Compounds Known to Be Sweet in Man. Food Chem..

[45] Zhang W., Heng J., Kim S.W., Chen F., Deng Z., Zhang S., Guan W. (2020). Dietary Enzymatically-Treated *Artemisia annua* L. Supplementation Could Alleviate Oxidative Injury and Improve Reproductive Performance of Sows Reared under High Ambient Temperature. J. Therm. Biol..

[46] Mayhew D.A., Phil Comer C., Wayne Stargel W. (2003). Food Consumption and Body Weight Changes with Neotame, a New Sweetener with Intense Taste: Differentiating Effects of Palatability from Toxicity in Dietary Safety Studies. Regul. Toxicol. Pharmacol..

[47] Kennedy J.M., Baldwin B.A. (1972). Taste Preferences in Pigs for Nutritive and Non-Nutritive Sweet Solutions. Anim. Behav..

[48] Roura E., Fu M. (2017). Taste, Nutrient Sensing and Feed Intake in Pigs (130 Years of Research: Then, Now and Future). Anim. Feed Sci. Technol..

[49] Tomita T., Sato N., Arai T., Shiraishi H., Sato M., Takeuchi M., Kamio Y. (1997). Bactericidal Activity of a Fermented Hot-Water Extract from *Stevia rebaudiana* Bertoni towards Enterohemorrhagic *Escherichia coli* O157:H7 and Other Food-Borne Pathogenic Bacteria. Microbiol. Immunol..

[50] Chatsudthipong V., Muanprasat C. (2009). Stevioside and Related Compounds: Therapeutic Benefits beyond Sweetness. Pharmacol. Ther..

[51] Daneshyar M., Geuns J.M.C., Willemsen H., Ansari Z., Darras V.M., Buyse J.G., Everaert N. (2012). Evaluation of Dietary Stevioside Supplementation on Anti-human Serum Albumin Immunoglobulin G, Alpha-1-glycoprotein, Body Weight and Thyroid Hormones in Broiler Chickens. J. Anim. Physiol. Anim. Nutr..

[52] Li S., Chen T., Dong S., Xiong Y., Wei H., Xu F. (2014). The Effects of Rebaudioside A on Microbial Diversity in Mouse Intestine. Food Sci. Technol. Res..

[53] Liao S.F., Nyachoti M. (2017). Using Probiotics to Improve Swine Gut Health and Nutrient Utilization. Anim. Nutr..

[54] Papaefthimiou M., Kontou P.I., Bagos P.G., Braliou G.G. (2023). Antioxidant Activity of Leaf Extracts from Stevia Rebaudiana Bertoni Exerts Attenuating Effect on Diseased Experimental Rats: A Systematic Review and Meta-Analysis. Nutrients.

[55] Tang M., Zhao J., Wu Y., Yu C., Peng C., Liu H., Cui Y., Lan W., Lin Y., Kong X. (2024). Improving Gut Functions and Egg Nutrition with Stevia Residue in Laying Hens. Poult. Sci..

[56] Wen K., Zhang K., Gao W., Bai S., Wang J., Song W., Zeng Q., Peng H., Lv L., Xuan Y. (2024). Effects of Stevia Extract on Production Performance, Serum Biochemistry, Antioxidant Capacity, and Gut Health of Laying Hens. Poult. Sci..

[57] Medeot D.B., Nilson A., Miazzo R.D., Grosso V., Ferrari W., Jofré E., Soltermann A., Peralta M.F. (2023). Stevia as a Natural Additive on Gut Health and Cecal Microbiota in Broilers. Vet. Anim. Sci..

[58] Del Pozo S., Gómez-Martínez S., Díaz L.E., Nova E., Urrialde R., Marcos A. (2022). Potential Effects of Sucralose and Saccharin on Gut Microbiota: A Review. Nutrients.

[59] Chen C.-Y., Tien C.-H., Chen Y.-H., Garrido D., Farzi A., Herzog H., Fan H.-Y., Chen Y.-C. (2024). Effect of Sucralose Intake on Human and Mouse/Rat Gut Microbiota Composition: A Systematic Review and Meta-Analysis. Food Rev. Int..

